# Effects of Sheep and Mouse Urine on the Growth Pattern of *Leishmania major* Promastigotes

**DOI:** 10.1155/2013/748592

**Published:** 2013-07-15

**Authors:** Vahid Nasiri, Gholamreza Karimi, Abdolhossein Dalimi, Habibollah Paykari, Fatemeh Ghaffarifar

**Affiliations:** ^1^Parasitology Department, Razi Vaccine and Serum Research Institute, P.O. Box 31975-148, Karaj, Alborz, Iran; ^2^Parasitology and Entomology Department, Faculty of Medical Sciences, Tarbiat Modares University, P.O. Box 14115-331, Tehran, Iran

## Abstract

The protozoan parasites of the genus *Leishmania* are the causative agents of different clinical diseases. Fetal calf serum (FCS) is the main part and the most expensive ingredient of the *Leishmania* culture media. Here, the efficacies of different concentrations (1%, 2.5%, 5%, and 10%) of the filtered and autoclaved sheep and mouse urine were evaluated as a growth stimulator in *Leishmania* culture procedure. The results indicated that culture media enriched with the filtered sheep and mouse urine supported the growth of the parasites and can be used for cultivation of *Leishmania* parasites. In conclusion, this study has demonstrated an alternative low-cost medium that could be used in cultivation process of *Leishmania major* promastigotes.

## 1. Introduction

Leishmaniasis is caused by parasitic protozoa transmitted by the bite of female sand fly, affecting 12 million people worldwide. Several species of *Leishmania* caused human diseases that range from self-healing cutaneous lesions to fatal visceral Leishmaniasis (VL), mucosal Leishmaniasis, and diffuse cutaneous Leishmaniasis [1]. This parasite is reported in 88 endemic countries, of which 82% are low-income countries [2], and is the cause of one of the 6 primary tropical diseases [3]. Globally, there are an estimated 1.5–2 million new cases and 70 000 deaths each year, and 350 million people are at risk of infection and disease. It causes an estimated 2.4 million disability-adjusted life years [4].

The many of different kinds of media that have been used for cultivation of genus *Leishmania*, the basic requirement for any research on the parasites, require fetal calf serum (FCS) as one of their essential ingredients. Using serum in cell culture is problematic for several reasons. Serum is a complex, highly variable, and difficult to characterize reagent [5]. FCS is highly expensive, and reliable supply is very difficult to obtain, especially in developing countries [6]. Developing serum-free media has been the philosopher's stone of cell culturists since animal cells were first cultured *in vitro* more than half a century ago [7]. Several attempts have been made to replace FCS with different kinds of different sera, bovine serum albumin, a mixture of purine bases, vitamins, large concentrations of certain amino acids, hormones, hemin, hemoglobin, human and animal urine, and, more recently chicken serum [8–16] introduced as an alternative low-cost serum that can be used in culture medium for primary isolation, routine cultivation, and mass cultivation of *Leishmania *parasites [16]. The utilization of sodium urate, uric acid, and cysteic acid, known components of the urine of the insect vector, for *in vitro* growth and differentiation of *Leishmania donovani* [17] has motivated the use of human urine as a constituent of *Leishmania *spp. culture media. A number of reports have shown that the addition of 1–5% human urine stimulates the growth of the promastigotes of leishmanian parasites, leading to a more rapid multiplication and a higher concentration of parasites at the stationary phase [5, 8, 13, 18–20]. 

In the present work, we evaluated the efficacy of the sheep and mouse urine as a growth stimulator and probability of replacing FCS with them and optimizing the urine-enriched medium for *Leishmania major *promastigotes culture procedure.

## 2. Material and Methods 

### 2.1. Urine Collection

Fresh sheep urine was obtained by intramuscular injection of furosemide as diuretic drug to a male sheep and about 200 mL of urine was collected after 10–15 minutes. 

By handling the outbred laboratory white mice they began to urinate, and then we collected this urine in some tubes. We obtained urines from both male and female mice.

These urines made sterile both by passing through 0.22 *μ*m membrane filter and by autoclaving at 121°C for 15 minute. All dilutions and origins of urines were used freshly and for comparisons some parts of them were stored by freezing at −20°C until use. 

### 2.2. Medium Preparation

RPMI-1640 was used as a standard basal medium for growth pattern evaluation and prepared by dissolving 1.04 g of RPMI-1640 (sigma) in 90 mL of distilled water and the pH was adjusted to 7 with 1 N NaOH/HCl solutions and then 0.2 g NaHCO_3_ added for fixing buffered condition. This medium was made sterilized by pressure passage through 0.22 *μ*m membrane filters (Millipore, Germany) and then 10 mL (10%) urine (filtered and autoclaved of both sheep and mice) was added. Four dilutions of urines were prepared as follows: 1%, 2.5%, 5%, and 10%. No antibiotic in culture media was used. Above procedures were used for preparation of RPMI-1640 enriched with (10%) heat-inactivated fetal calf serum instead of urine for positive control. The complete media were kept at 4°C until use.

### 2.3. Parasite Cultivation

*Leishmania *promastigotes (*Leishmania major*: MRHO/IR/76/ER) 2nd subculture that previously had been grown in RPMI-1640 medium supplemented with 10% FCS, were concentrated by centrifugation at 2500 g for 10 minutes and washed twice with sterile phosphate-buffered saline solution (PBSS) to remove any traces of FCS. Parasites were counted by using a Neubauer chamber (haemocytometer) slide under invert microscopy and diluted in PBSS. Subcultures in different media were prepared from these washed and diluted promastigotes. On the whole subcultures were performed in 5 repeated series and in each series, triplicate cultures of media were prepared for each origin and dilution of urines. Complete media (enriched with 1%, 2.5%, 5%, and 10% filtered/autoclaved urine of sheep/mouse) alongside the positive control media (enriched with 10% FCS) were inoculated with midlog phase promastigotes in 25 cm^2^ plastic culture flasks at the final concentration of 5 × 10^6^ promastigotes/mL. On the whole every flask contained 10 mL of mixture of promastigotes and media for each of culture conditions. The flasks were placed in incubator at 26°C and continuous cultures of the *parasites *were made by subculturing into fresh medium (at day 5 after every passage) weekly until they reached 10th subculture. In all subcultures, parasites growth was assessed qualitatively and quantitatively by microscopic observations and the Giemsa slide preparation, and the number of parasites was counted every day using haemocytometer slide. 

### 2.4. *In Vivo* Determination of Parasites Infectivity

3 × 10^6^ parasites from stationary-phase promastigotes of *Leishmania major* of all growth supported culture media from 5th subculture were washed twice in sterile phosphate-buffered saline solution (PBSS) and were inoculated subcutaneously into the base of tail of 5 male BALB/c mice (6-7 weeks old), with five mouse serving as negative control. The presences of typical lesions were evaluated macroscopically every day and after appearing the lesion biopsy was performed and inoculated in NNN medium.

### 2.5. Statistical Evaluation

SPSS-18 for windows were used to evaluate the data. The differences between the averages of the quantitative variables were evaluated by Student's *t*-test and the value of *P* < 0.05 was accepted as statistically significant.

## 3. Results

### 3.1. Assessment of Parasite Growth Quality and Replication Pattern

**Figure 1:**
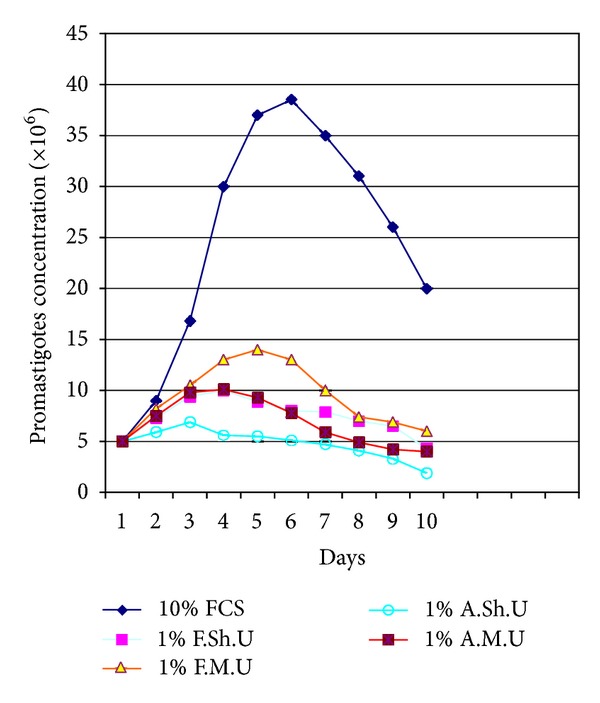
Growth curve of *Leishmania major *promastigotes in RPMI-1640 medium plus 10% fetal calf serum (FCS) and RPMI-1640 medium plus 1% filtered sheep urine (F.Sh.Ur), filtered mouse urine (F.M.Ur), autoclaved sheep urine (A.Sh.Ur), and autoclaved mouse urine (A.M.Ur).

**Figure 2:**
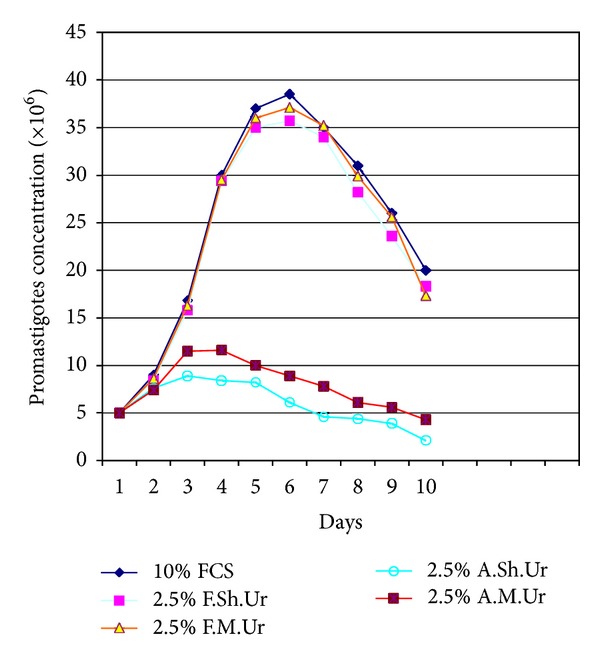
Growth curve of *Leishmania major *promastigotes in RPMI-1640 medium plus 10% fetal calf serum (FCS) and RPMI-1640 medium plus 2.5% filtered sheep urine (F.Sh.Ur), filtered mouse urine (F.M.Ur), autoclaved sheep urine (A.Sh.Ur), and autoclaved mouse urine (A.M.Ur).

**Figure 3:**
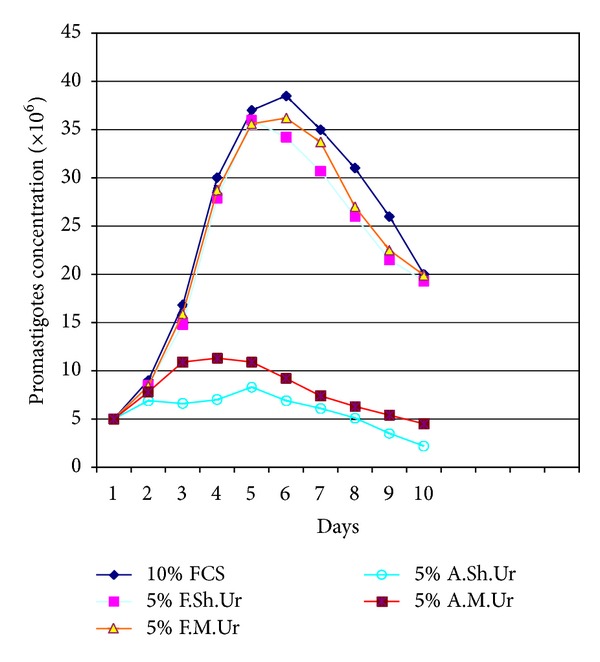
Growth curve of *Leishmania major *promastigotes in RPMI-1640 medium plus 10% fetal calf serum (FCS) and RPMI-1640 medium plus 5% filtered sheep urine (F.Sh.Ur), filtered mouse urine (F.M.Ur), autoclaved sheep urine (A.Sh.Ur), and autoclaved mouse urine (A.M.Ur).

**Figure 4:**
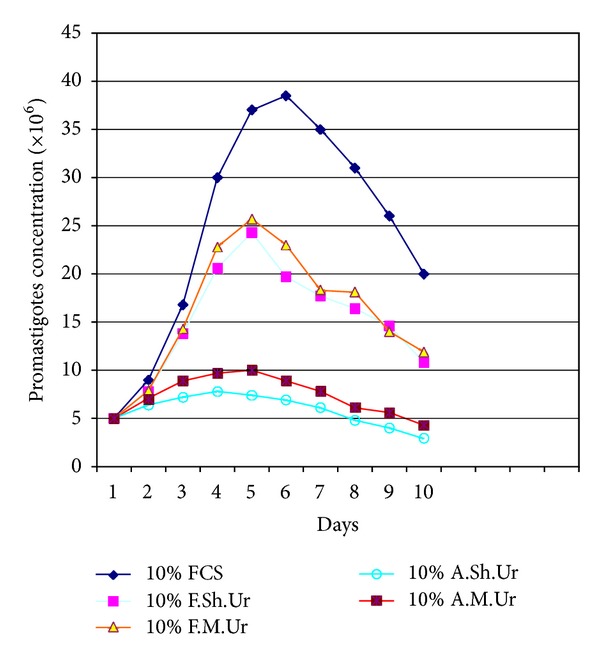
Growth curve of *Leishmania major *promastigotes in RPMI-1640 medium plus 10% fetal calf serum (FCS) and RPMI-1640 medium plus 10% filtered sheep urine (F.Sh.Ur), filtered mouse urine (F.M.Ur), autoclaved sheep urine (A.Sh.Ur), and autoclaved mouse urine (A.M.Ur).

Results indicated that the addition of 2.5–5% filtered sheep or mouse urine to RPMI-1640 medium significantly stimulated the growth of the promastigotes of *Leishmania major*, when compared with the parasite growth in conventional RPMI-1640 medium enriched with fetal calf serum (FCS), and it was equivalent to the growth observed in RPMI-1640 supplemented with FCS (*P* < 0.05). Fine grown parasites and typical morphology of promastigotes were observed in the Giemsa-stained smears prepared from the culture media containing the suitable dilutions (2.5–5%) of urines (filtered urine of sheep and mouse). Under invert microscopy it was observed that the promastigotes were elongate and had rapid motility and made rosette forms that indicate appropriate culture conditions. It was observed that the culture medium containing suitable dilutions (2.5–5%) of urines (filtered urine of sheep and mouse) supported the continuity of the parasites in successive passages. When filtered and autoclaved urines were used, in all dilution, it was observed that there are many quality and quantity differences in *L. major* promastigotes growth pattern (*P* < 0.05). The comparison curves of the parasites growth in different dilutions and kinds of urines were shown in Figures 1, 2, 3, and 4.

### 3.2. Effect of Urine Percentage on Growth Quantity of Promastigotes

Inoculated parasites into the culture media that contained appropriate dilutions (2.5–5%) of urines (filtered urine of sheep and mouse) took about 5-6 days to reach the late log phase. The growth of the parasites was maximally enhanced by 2.5% of filtered sheep urine. We have not seen any difference between fresh and stored urines at −20°C in ability to supporting growth pattern of promastigotes.

### 3.3. *In Vivo* Assessment of Pathogenicity

Three to five weeks following inoculation of the parasites from 5th subcultures containing 2.5% filtered urine of mouse and sheep to BALB/c mice, the presence of typical cutaneous lesions of Leishmaniasis were observed on the base of the tail of all experimented animals. By sampling from the lesions and transferring the exudates to RPMI-1640 medium enriched with 2.5% filtered urine of mouse or sheep and subculturing the resulting promastigotes, we were able to confirm that these enriched media could support transformation of amastigotes of *Leishmania* from skin lesion into promastigotes, indicating that the medium can be used for parasites isolation from infected samples.

## 4. Discussion

The cell culture technique is an approach to make efforts to prepare complex habitat conditions of living organisms to develop our knowledge about their behavior and find suitable ways (like effective vaccine) to prevent negative side effects of them. Leishmaniasis is a major tropical disease acknowledged by WHO [3] and for many years making an effective vaccine remained as a dreamy desire especially for tropical and subtropical countries where the disease is a result of poverty and poor healthy conditions. On the other hand, routine commercially culture media for cultivation of *Leishmania* such as RPMI-1640, medium 199, and Schneider's *Drosophila *that were enriched by FCS or blood lysate [21, 22] are very expensive. The major problem for mass cultivation of Leishmania parasites is the need to FCS that its quality varies in each production and is not manufactured in many countries, specially most of the poor tropical countries, that often Leishmaniasis is one of the major health problems of them.

Urine is a complex mixture, making difficult the characterization of the components which are capable of promoting the growth of some organisms including the Trypanosomatidae family. Ferreira et al. [23] showed that the addition of 3% human urine to LIT medium significantly stimulated the growth of all the *T. rangeli* strains equivalently to the FCS-enriched medium. They mentioned that the use of human urine in culture media for *T. cruzi* and *T. rangeli* shows several advantages, such as the availability and absence of costs in obtaining the urine samples and the lack of inhibitory effects in parasite growth. In their experiments, the ability of human urine to promote *in vitro* growth has not been lost by the heat denaturation of the human urine sample [23]. Initial researches have indicated that the factor responsible for this enhancement is a small molecule which is not destroyed by autoclaving [8]. In contrast with this researches our data imply appreciable difference between filtered and autoclaved urine. Studies have shown that promastigotes of various species of *Leishmania *can use several carbohydrates as respiratory substrate including glucose, fructose, mannose, and galactose [24]. Some studies demonstrated the role of amino acids as primary growth substrate [25, 26]. In a study it has been showed that like 5% (v/v) human urine, 10 *μ*M xanthine significantly enhances the growth of *L. major* promastigotes in culture, suggesting that the xanthine is the most active molecule in human urine that enhances the growth of *L. major* promastigotes in culture, and its routine addition at 10 *μ*M should improve the culture of *Leishmania in vitro* [27]. Urine contains significant amounts of both nitrogen and carbon, and typically 70% of urine N is urea and the rest consists of amino acids (principally glycine) and peptides [28]. Amino acids such as glycine and amines such as creatinine are components of sheep urine [29]. Parasites of the genus *Trypanosoma* possess a receptor for the epidermal growth factor (EGF), a protein usually found in human urine [30] and implicated as the factor that would lead to parasite growth *in vitro* [23]. Another possible candidate is biopterin, an enzymatic cofactor known to be essential for kinetoplastid growth [31] and found in approximate concentrations of 6.7 nM in samples of human urine from healthy individuals [32]. It is observed that dated urine has less stimulatory effect on the growth of the parasites as compared to fresh urine. The effect of the urine of different groups is also observed and it is found that the urine of 60 years old gives maximum growth and it is also found that female urine supported comparatively better growth as compared to male urine [20]. Several studies have been conducted in animal *Leishmania* that indicated leishmaniasis can affect horses and this point was documented in Spain and Portugal upon detection of *L. infantum* in this host [33–35]. Recently, an accumulation of sporadic cases of cutaneous leishmaniasis has been documented in horses originating from Germany and Switzerland [36]. One case report described a novel etiological agent of cutaneous leishmaniasis that appears for the first time in a cow [37]. Another research founded infections among persons (6.1%), cows (5%), buffaloes (4%), and goats (16%) [38]. Intracellular amastigote-like bodies have been reported in a biopsy section from the pinna of a sheep in South Africa [39]. In a study, the canine urine with optimum concentration about 8% supported similar levels of parasite multiplication to those seen with 5% healthy-human urine and the maximum concentrations of parasites seen in the trials with the bovine urine were observed when the urine was present at 10% but these were significantly lower than those seen with 8% canine urine [13]. This study mentioned that higher concentrations of urine were not as good, probably because of a fall in pH and inhibitory concentrations of human metabolites [13]. But a research reported that human urine in parasite cultures differentially affected and increased the infectivity and proliferation of *Leishmania *species promastigotes and facilitated the transition from G0/G1 to S phases more quickly and their results have shown that promastigotes could efficiently proliferate and grow in culture media containing relatively high (25%) urine concentrations [40]. In our previous researches about finding suitable replacements for FCS we had found that the serum and the urine of many animals like hamster, rabbit, and sheep are suitable for cultivation of promastigotes, but, collecting the serum of some of them, like Hamster, is expensive and using of some of them, like sheep serum, is accompanied with many challenges about adaptation problems of promastigotes to new serum (unpublished work). Our recent study about finding replacement for FCS in cultivation of promastigotes of *Leishmania infantum* indicated that chicken serum is very suitable for nutritional requirement of parasites and is a comparatively simply available and inexpensive serum that can replace in the media that required FCS enhancement for promastigote forms and indicate a potentiality of the new medium to be used in long-term *in vitro* cultivation of *Leishmania *promastigotes [41]. But we are searching to find novel stimulating factors to promoted growth pattern of parasites that could be prepared chemically and we believe that urine could lead us to find these unknown materials. 

## 5. Conclusion

Sheep urine is suitable for nutritional requirement of parasites and is readily available at low cost and preparation of it does not require sophisticated expensive equipment and can be collected from every place even in poor countries. The BALB/c mice are available in all of the laboratories and their urine could be collected for small scale purpose of *Leishmania *cultivation. They could be replaced in the media that require FCS enhancement and the new medium could be used for *in vitro* cultivation of *Leishmania* promastigote. This study has demonstrated an alternative low-cost medium that could be used in cultivation process of promastigotes of leishmanian parasites especially in poor countries.
